# Metformin Enhancement of Therapeutic Effects of 5-Fluorouracil and Oxaliplatin in Colon Cancer Cells and Nude Mice

**DOI:** 10.3390/biomedicines10050955

**Published:** 2022-04-20

**Authors:** Kwan-Ling Yip, Tsen-Ni Tsai, I-Ping Yang, Zhi-Feng Miao, Yen-Cheng Chen, Ching-Chun Li, Wei-Chih Su, Tsung-Kun Chang, Ching-Wen Huang, Hsiang-Lin Tsai, Yung-Sung Yeh, Jaw-Yuan Wang

**Affiliations:** 1 Division of Colorectal Surgery, Department of Surgery, Kaohsiung Medical University Hospital, Kaohsiung Medical University, Kaohsiung 80708, Taiwan; kwanyhk@hotmail.com (K.-L.Y.); jennytsai1978@yahoo.com.tw (T.-N.T.); zfmiao@yahoo.com (Z.-F.M.); googoogi05@gmail.com (Y.-C.C.); dobird05@yahoo.com.tw (C.-C.L.); lake0126@yahoo.com.tw (W.-C.S.); tsungkunchang@gmail.com (T.-K.C.); baseball5824@yahoo.com.tw (C.-W.H.); chunpin870132@yahoo.com.tw (H.-L.T.); 2 Department of Nursing, Shu-Zen College of Medicine and Management, Kaohsiung 82144, Taiwan; yangiping000@gmail.com; 3 Graduate Institute of Clinical Medicine, College of Medicine, Kaohsiung Medical University, Kaohsiung 80708, Taiwan; 4 Department of Surgery, Faculty of Medicine, College of Medicine, Kaohsiung Medical University, Kaohsiung 80708, Taiwan; 5 Division of Trauma and Surgical Critical Care, Department of Surgery, Kaohsiung Medical University Hospital, Kaohsiung Medical University, Kaohsiung 80708, Taiwan; yossel.yeh@msa.hinet.net; 6 Department of Emergency Medicine, Faculty of Post-Baccalaureate Medicine, College of Medicine, Kaohsiung Medical University, Kaohsiung 80708, Taiwan; 7 Graduate Institute of Medicine, College of Medicine, Kaohsiung Medical University, Kaohsiung 80708, Taiwan; 8 Center for Cancer Research, Kaohsiung Medical University, Kaohsiung 80708, Taiwan; 9 Center for Liquid Biopsy and Cohort Research, Kaohsiung Medical University, Kaohsiung 80708, Taiwan; 10 Pingtung Hospital, Ministry of Health and Welfare, Pingtung 90054, Taiwan

**Keywords:** metformin, colorectal cancer, 5-fluorouracil/oxaliplatin, chemotherapy

## Abstract

Studies have demonstrated that metformin has antitumor effects in addition to therapeutic effects on hyperglycemia; however, few studies have explored the effects of metformin in chemotherapy. Therefore, we hypothesized that the administration of metformin would enhance the therapeutic effects of 5-fluorouracil and oxaliplatin (FuOx) to inhibit the growth of colorectal cancer (CRC) cells in vitro and in vivo. The results of our in vitro experiments demonstrated that metformin significantly increased the effects of FuOx with respect to cell proliferation (*p* < 0.05), colony formation (*p* < 0.05), and migration (*p* < 0.01) and induced cell cycle arrest in the G0/G1 phase in HT29 cells and the S phase in SW480 and SW620 cells (*p* < 0.05). Flow cytometry analysis revealed that metformin combined with FuOx induced late apoptosis (*p* < 0.05) by mediating mitochondria-related Mcl-1 and Bim protein expression. Furthermore, in vivo, metformin combined with FuOx more notably reduced tumor volume than FuOx or metformin alone did in BALB/c mice (*p* < 0.05). These findings demonstrate that metformin may act as an adjunctive agent to enhance the chemosensitivity of CRC cells to FuOx. However, further clinical trials are warranted to validate the clinical implications of the findings.

## 1. Introduction

Colorectal cancer (CRC) is one of the most prevalent malignancies worldwide. According to data from a 2020 statistical analysis, globally, CRC accounts for 10% of all incident cancers (1.9 million cases) in both sexes. Furthermore, CRC has the second highest mortality rate among incident cancers, comprising 9.4% of total cancer deaths (0.93 million cases) [1]. The CRC incidence rate has gradually increased in Taiwan [2]. CRC is the most common cancer in men and the second most common cancer in women, with age-standardized incidence rates of 52.26 and 34.61 per 100,000 persons, respectively, in 2019. CRC is also the third leading cause of cancer-related death, with incident rates of 18.4 and 11.56 per 100,000 persons in men and women, respectively.

The pyrimidine analog 5-fluorouracil (5-FU) is widely used in combination with other chemotherapy drugs, such as oxaliplatin, to treat a range of cancers, including breast, gastric, pancreatic, colorectal, and head and neck cancers [3]. Moreover, 5-FU has long been essential in CRC chemotherapy. Fluorouracil-based chemotherapy, FOLFOX (5-FU, leucovorin, and oxaliplatin), is a standard adjuvant chemotherapy regimen in patients with stage III CRC [4]. Employing postoperative adjuvant chemotherapy can reduce patients’ chemoresistance or recurrence to improve their survival rates [5,6]. Therefore, developing a novel adjuvant drug is vital to achieve favorable outcomes and minimize the occurrence of adverse events.

Clinicians are increasingly adding adjuvant drugs to conventional chemotherapy regimens to promote the therapeutic efficacy [7,8]. Metformin (1,1-dimethylbiguanide hydrochloride), which is a biguanide, is the most widely used oral antidiabetic drug for treating type 2 diabetes [9]. Several clinical trials have investigated the association between metformin and cancer in patients with diabetes and have demonstrated that patients treated with metformin had lower cancer incidence and mortality rates than patients not treated with metformin [10,11,12]. Our previous study also revealed that metformin can delay the development of CRC in a dose-dependent manner in patients with type 2 diabetes mellitus (T2DM) [13]. In addition, epidemiological studies have revealed that patients with T2DM and cancer who were treated with metformin demonstrated more significant improvement in progression-free survival and overall survival than patients who received other antidiabetic drugs [14,15,16]. Metformin has been reported to suppress the proliferation of cancer cells [17,18] and thereby enhance the anticancer effects of chemotherapy in various cancers, such CRC, cervical cancer, and leukemia [19,20,21]. Therefore, experiments exploring the potential correlation between metformin use and CRC development and examining the role of metformin in CRC therapy are gaining attention.

An accumulating body of evidence has indicated that B-cell lymphoma (Bcl-2) family proteins are involved in the regulation of apoptosis [22]. Myeloid cell leukemia 1 (Mcl-1) and Bcl-2-like protein 11 (Bim), which are members of the Bcl-2 protein family, play a critical role in mitochondrial-mediated apoptosis. The antiapoptotic protein Mcl-1 interacts with other Bcl-2 family members, such as the proapoptotic protein Bim, in the mitochondria to initiate apoptosis. In a review article, Belmar et al., revealed that Mcl-1 was highly expressed in breast cancer, pancreas cancer, and leukemia, which enable cancer cells to develop resistance against chemotherapy agents [23]. However, the association between Mcl-1 expression and therapeutic strategies in CRC is unclear.

Based on the aforementioned assumption, we evaluated whether the proliferation of CRC cells would be further diminished when additional metformin was administered with chemotherapeutic agents. In this study, we aim to verify that metformin can enhance the therapeutic effects of 5-FU and oxaliplatin (FuOx) on CRC cells in vitro and in vivo and further elucidate the mechanism underlying metformin enhancing the effects of FuOx.

## 2. Materials and Methods

### 2.1. Drugs

Metformin (#D150959), 5-FU (#F6627), and leucovorin (#F7878) were purchased from Sigma-Aldrich (St. Louis, MO, USA). Metformin was dissolved in phosphate-buffered saline (PBS) to prepare a 1 M stock solution. In addition, 5-FU and leucovorin were diluted in PBS to obtain 5 mg/mL stock solution. Oxaliplatin was obtained from Sanofi (Sanofi, Gentilly, France). All drugs were filtered through a 0.22 µM pore filter before use in the experiment.

### 2.2. Cell Culture

An HT29 colon cancer cell line was purchased from American Type Culture Collection (Manassas, VA, USA), and SW480 and SW620 colon cancer cell lines were purchased from the Bioresource Collection and Research Center (Hsinchu, Taiwan). All cell lines were maintained in Dulbecco’s Modified Eagle Medium (Gibco, Grand Island, NY, USA) supplemented with 4.5 g/L D-glucose, 10% fetal bovine serum (Gibco), and 1% penicillin/streptomycin (Gibco) in a 37 °C incubator with 5% CO_2_.

### 2.3. Cell Counting Kit-8 (CCK-8) Assay

The HT29 (8 × 10^3^ cells/well), SW620, and SW480 (7 × 10^3^ cells/well) cells were seeded in 96-well plates. After 24 h of adherence, the cells were treated with various concentrations of metformin (0–100 mM), 5-FU (0–100 µM), and oxaliplatin (0–100 µM) for 24, 48, and 72 h. Furthermore, the cells were treated with FuOx and the combination of metformin and FuOx for an additional 72 h, followed by the addition of 10 µL CCK-8 reagent (Sigma-Aldrich, St. Louis, MO, USA) for 3 h at 37 °C. Absorbance was measured at 450 nm using a microplate reader (BioTek Instrument, Winooski, VT, USA).

### 2.4. Colony Formation Assay

The colon cells were seeded in 6-well plates at a density of 3 × 10^3^ cells in each well for 24 h. Subsequently, the cells were incubated with metformin, FuOx, and the combination of metformin and FuOx. After 1 week of growth, the cells were washed twice with PBS, fixed with 4% paraformaldehyde, and stained with crystal violet. The data were quantified using ImageJ software (Java 1.8.0_172; National Institutes of Health, Bethesda, MD, USA).

### 2.5. Wound-Healing Assay

We adjusted the cell suspension to a concentration of 5 × 10^5^ cells/mL and added 80 µL into each well of the culture-inserts (ibidi GmbH, Gräfelfing, Germany). After the cells formed a monolayer, the culture-inserts were removed. The cells were washed with PBS and cultured in the presence of metformin, FuOx, and the combination of metformin and FuOx. The migrated cells were photographed with Leica DMI6000 B (Leica Microsystems, Wetzlar, Germany) at various time points (24, 48, 72, and 96 h). The area of wound closure was analyzed using Image J software (National Institutes of Health).

### 2.6. Cell Cycle Analysis

The cells (1 × 10^6^) were plated in 6-well plates overnight and were then incubated with serum-free medium for an additional 24 h. The flesh culture media containing metformin, FuOx, and the combination of metformin and FuOx were added. After 24 h, the cells were harvested and washed once with PBS and were permeabilized with 5 mL cold 75% ethanol at −20 °C overnight. The fixed cells were centrifuged and washed twice with PBS. The cells were stained with 0.3 mg/mL RNase A, 0.2% Triton-X 100, and 30 µg/mL propidium iodide (Sigma-Aldrich) in 1 mL of PBS and were placed in the dark for at least 30 min. The stained cells were analyzed using the FC500 flow cytometer (Beckman Coulter, Brea, CA, USA).

### 2.7. Apoptosis Analysis

The cells (7 × 10^3^) were plated in 6-well plates and, after 24 h, were treated with metformin, FuOx, and the combination of metformin and FuOx for 72 h. The cells were collected, centrifuged, and rinsed twice with PBS. The cells were then resuspended in 500 µL 1× binding buffer and were stained with 5 µL PE Annexin V and 7-AAD (BD Bioscience, Franklin Lakes, NJ, USA) at room temperature in the dark for 20 min. The stained cells were analyzed using flow cytometry FC500 (Beckman Coulter).

### 2.8. Western Blotting

The cells were harvested after treatment with metformin, FuOx, and the combination of metformin and FuOx for 72 h and were then lysed in a radioimmunoprecipitation assay (RIPA) buffer (Merck Millipore, Burlington, MA, USA) containing protease inhibitors (Sigma-Aldrich) and a phosphatase inhibitor cocktail (Sigma-Aldrich). The protein concentrations were quantitated using Protein Assay Dye (Bio-Rad Laboratories, Hercules, CA, USA). The protein samples (20 µg) were loaded and separated using SDS-PAGE and were transferred to polyvinylidene difluoride membranes (Merck Millipore). After the membranes were blocked, they were incubated with primary antibodies, namely Mcl-1 (1:1000; Cell Signaling Technology, Danvers, MA, USA), Bim (1:1000; Cell Signaling Technology), and β-actin (1:1000; Abcam, Cambridge, UK), at 4 °C overnight. The membranes were washed with tris-buffered saline (TBST) and incubated with secondary antibodies for 1 h at room temperature. The membranes were rinsed five times with TBST, and the bands were visualized through enhanced chemiluminescence detection reagent (Thermo Fisher Scientific, Waltham, MA, USA) using a ChemiDoc MP Imaging System (Bio-Rad Laboratories).

### 2.9. In Vivo Experiments

Seven-week-old male BALB/c nude mice were purchased from BioLasco Taiwan (Taipei, Taiwan). The mice were maintained in a pathogen-free environment and were randomly divided into four treatment groups: (I) control, (II) metformin-only, (III) FuOx-only, and (IV) combination of metformin and FuOx. After 1 week of acclimation, the mice were subcutaneously injected with HT29 cells (2 × 10^6^ cells/100 µL/mouse) on the right side of the back. Treatment was started 7 days after HT29 cell injection. The dose and schedule for metformin and FuOx administration were based on those in the literature [24,25,26]. Metformin (150 mg/kg) dissolved in drinking water was orally administered daily. The mice were injected with 6 mg/kg intraperitoneal oxaliplatin and 2 h later with 50 mg/kg 5-FU and 90 mg/kg leucovorin once a week for 4 weeks. Tumor diameters were measured three times per week, with tumor volume (mm^3^) calculated as volume = [length × width^2^]/2. The mice were sacrificed under anesthesia with isoflurane and cardiac puncture 1 week after the last chemotherapy treatment. The protocols of this in vivo study were approved by the Institutional Animal Care and Use Committee of Kaohsiung Medical University (IACUC Approval No.: 105074), and this study was conducted in accordance with the guidelines of the Care and Use of Laboratory Animals.

### 2.10. Statistical Analysis

All experiments were repeated in triplicate. Data are presented as mean ± standard error of the mean (SEM). Data were analyzed using one-way analysis of variance using Prism 9.0 software (GraphPad Software, San Diego, CA, USA), with *p* < 0.05 considered statistically significant.

## 3. Results

### 3.1. Metformin Increased the Inhibitory Effect of FuOx on Cell Proliferation, Colony Formation, and Cell Migration

**Figure 1:**
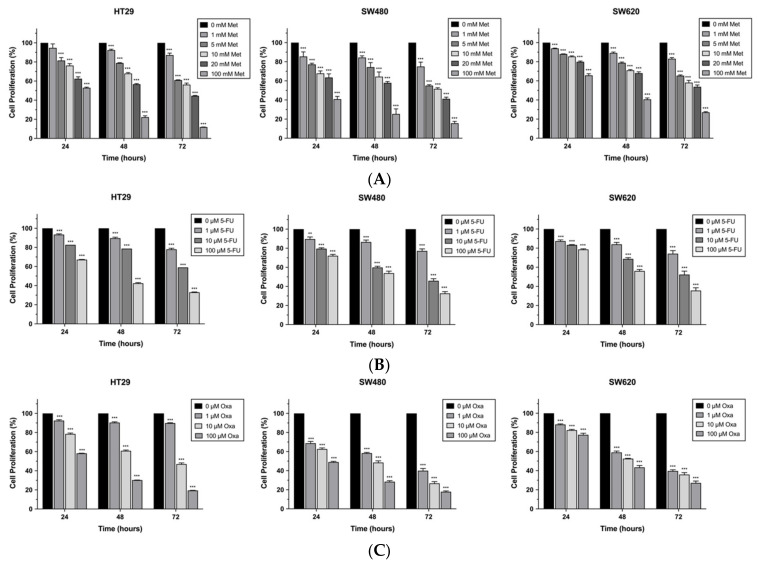
Metformin, 5-fluorouracil (5-FU), and oxaliplatin (Oxa) inhibit cell proliferation in CRC cell lines in dose-dependent and time-dependent manner. Cell proliferation detected at different time points after treatment with different doses and times for (**A**) metformin, (**B**) 5-FU, and (**C**) Oxa; data presented as mean ± standard error of the mean (SEM); ** *p* < 0.01, *** *p* < 0.001.

To assess the antiproliferation effect of metformin, 5-FU, and oxaliplatin on colon cancer cells, we treated HT29, SW480, and SW620 cells with various concentrations of metformin (0–100 mM), 5-FU (0–100 µM), and oxaliplatin (0–100 µM) for 24, 48, and 72 h. The results verified that all of the drugs could decrease the proliferation of the HT29, SW480, and SW620 cells in a dose-dependent and time-dependent manner (Figure 1).

**Figure 2:**
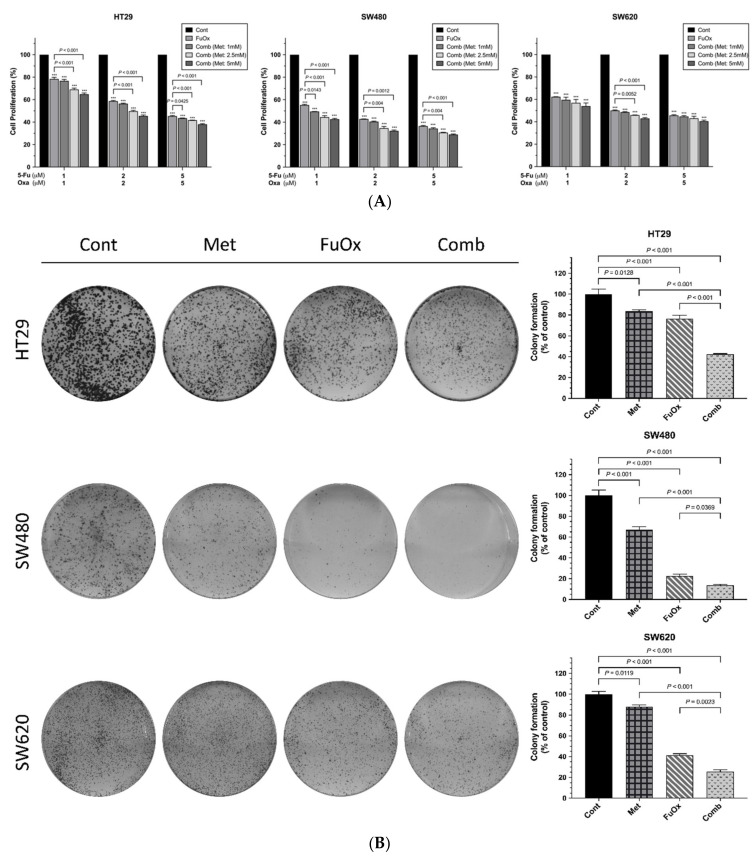

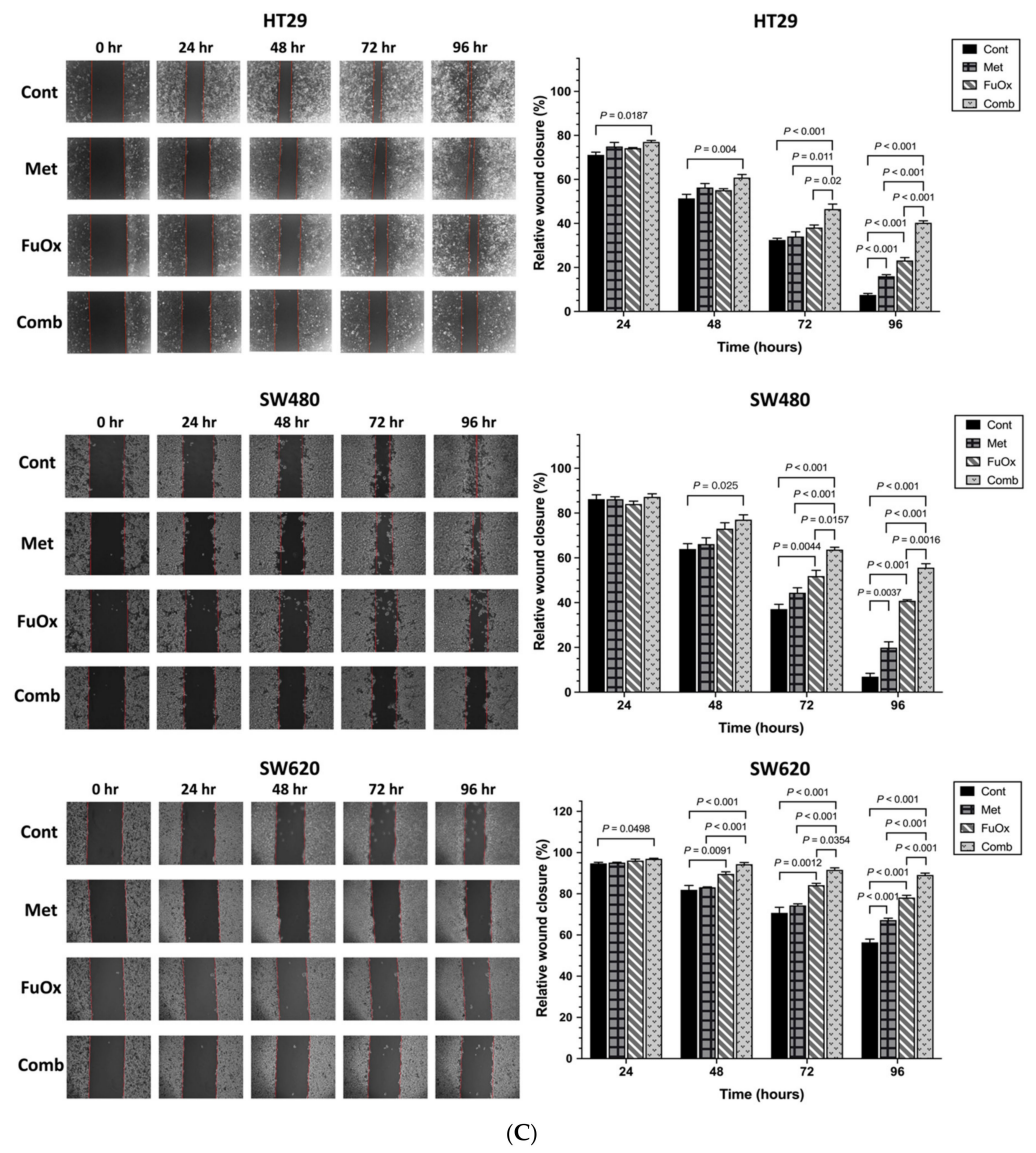
Metformin increases FuOx inhibition effect on cell proliferation, clonogenic growth, and migration. (**A**) Cell proliferation was measured using CCK-8 assay after cells were treated with the indicated concentrations of FuOx with or without metformin for 72 h; (**B**) cells were plated in 6-well plates at a density of 10,000 cells per well and were treated with metformin (5 mM), FuOx (2 µM), and the combination of metformin and FuOx. The number of colonies was counted after 7 days of treatment; (**C**) for migration assay, cells were added into each well of the 2-well culture-inserts in 12-well plates. When the cells had grown to 95% confluence, the 2-well culture-inserts were removed, and the cells were incubated in a medium with drugs for 96 h. Data are presented as mean ± SEM; *** *p* < 0.001. Cont, control; Met, metformin; FuOx, 5FU and oxaliplatin; Comb, combination of metformin and FuOx.

We further investigated cell proliferation under the combination of metformin and FuOx at 72 h. As presented in Figure 2A, compared with the control cells, the proliferation of the HT29 cells was significantly reduced by 22%, 41%, and 55% after treatment with 1, 2, and 5 µM FuOx (all *p* < 0.001) and was inhibited by 35%, 55%, and 65% when treated with 5 mM metformin plus 1, 2, and 5 µM FuOx, respectively (all *p* < 0.001). Additionally, compared with the control cells, the SW480 cell proliferation markedly decreased by 45%, 57%, and 63% after treatment with 1, 2, and 5 µM FuOx, respectively (all *p* < 0.001), and, respectively, decreased by 57%, 68%, and 71% after treatment with 5 mM metformin plus 1, 2, and 5 µM FuOx (all *p* < 0.001). Similarly, the SW620 cell proliferation significantly reduced by 38%, 50%, and 54% after, respectively, treatment with 1, 2, and 5 µM FuOx (all *p* < 0.001). After treatment with 5 mM metformin combined with 1, 2, and 5 µM FuOx, the SW620 cell proliferation, respectively, decreased by 46%, 57%, and 59% compared with the control cells (all *p* < 0.001). Therefore, the combination of metformin and FuOx was more effective in reducing the cell proliferation of the HT29, SW480, and SW620 CRC cell lines relative to the control cells and the cells treated with FuOx.

To investigate the colony formation ability of the CRC cell lines treated with the combination of metformin and FuOx, the CRC cells were incubated with metformin, FuOx, and the combination of metformin and FuOx for 1 week. The clonogenic capacity of the HT29, SW480, and SW620 cells treated with metformin or FuOx alone was lower than that of the control cells (all *p* < 0.05, Figure 2B). Moreover, the combination of metformin and FuOx notably elevated the inhibition of colony formation compared with the control and individual treatments (all *p* < 0.05). These results demonstrate that metformin can enhance the ability of FuOx to inhibit clonogenic growth.

As presented in Figure 2C, we also analyzed the effects of metformin and FuOx on the migration ability of CRC cells through a wound-healing assay. After 96 h of treatment, the migration rate was remarkably suppressed in the three cell lines treated with metformin or FuOx (all *p* < 0.01). Furthermore, the combination of metformin and FuOx inhibited the migration rate to a greater extent than metformin or FuOx alone did (all *p* < 0.01). In summary, metformin combined with FuOx significantly increased the inhibition of cell proliferation, colony formation, and migration, and the inhibition was notably more favorable than that of metformin or FuOx alone.

### 3.2. The Combination of Metformin and FuOx Induced Cell Cycle Arrest

**Figure 3:**
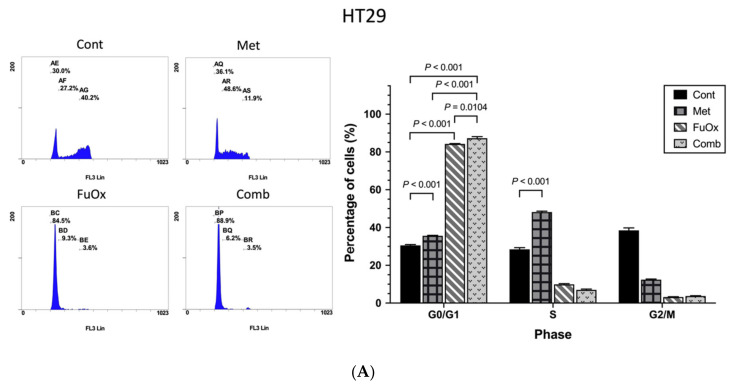

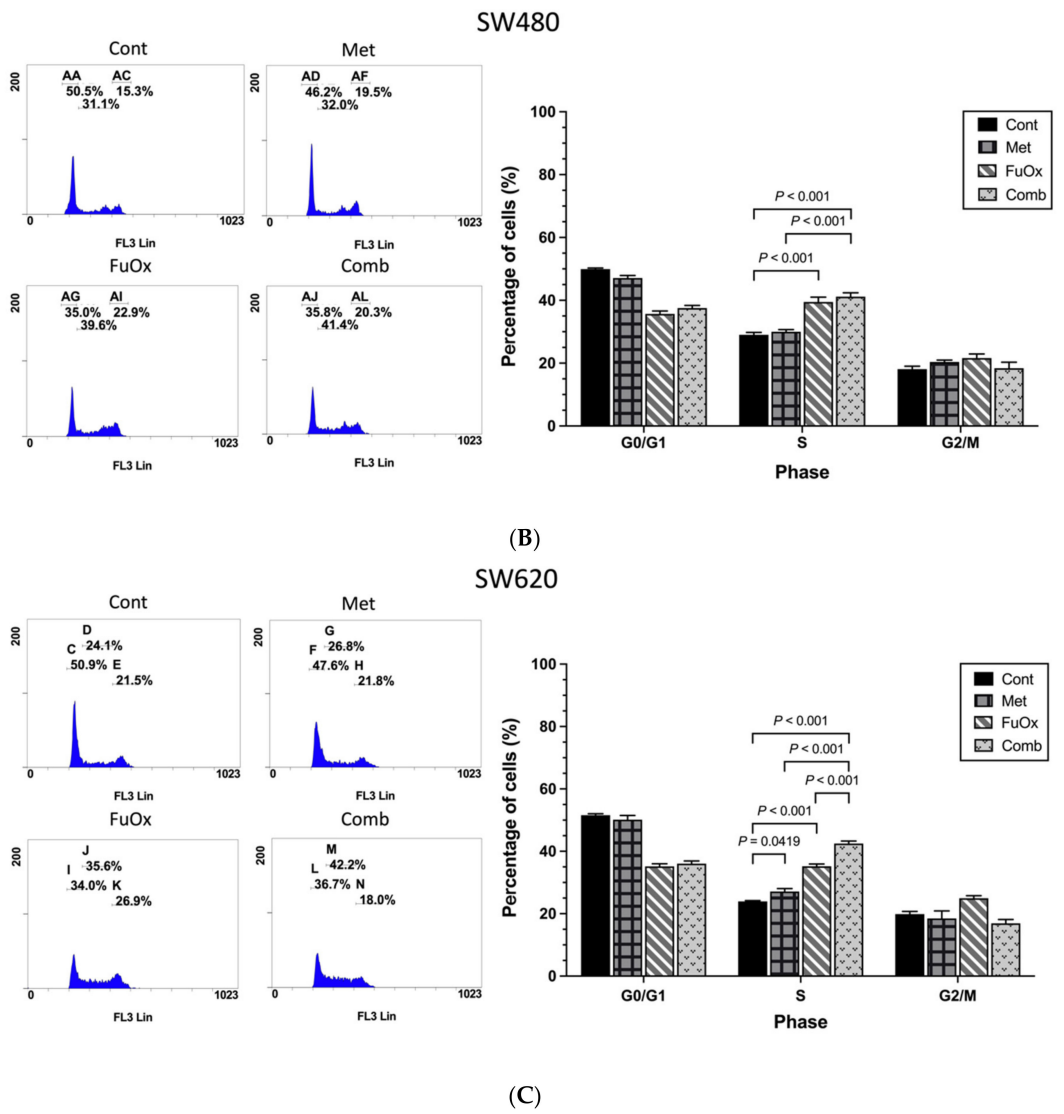
Combination therapy led to cell arrest in different phases in HT29, SW480, and SW620. CRC cells were analyzed using flow cytometry after 24 h of treatment with metformin (5 mM), FuOx (2 µM), and the combination of metformin and FuOx. Representative flow cytometry results (**left** panel) and histograms (**right** panel) reveal cell cycle distribution and quantitative analysis, respectively, in (**A**) HT29, (**B**) SW480, and (**C**) SW620 cell lines. Data are presented as mean ± SEM. Cont, control; Met, metformin; FuOx, 5FU and oxaliplatin; Comb, combination of metformin and FuOx.

To study the effect of metformin combined with FuOx on cell cycle distribution, cell cycles were analyzed using flow cytometry. In the HT29 cells, metformin-induced cell cycle arrest in the G0/G1 and S phases, whereas FuOx induced cell cycle arrest in the G0/G1 phase (all *p* < 0.001, Figure 3A). The combination of metformin and FuOx markedly induced more cell arrest in the G0/G1 phase than the control, metformin alone, and FuOx alone treatments, and cell cycle arrest was accompanied by a decrease in the cell population in the S phase (all *p* < 0.05, Figure 3A).

As displayed in Figure 3B, the cell cycle distribution in the SW480 cells treated with metformin alone showed no statistically difference (all *p* > 0.05). Although treatment with FuOx increased the percentage of cells in the S phase (*p* < 0.001), compared with FuOx treatment, metformin combined with FuOx did not significantly increase the proportion of cells in the S phase (*p* = 0.7009). In the SW620 cells, the increase in the cell proportion in the S phase in the combination group was prominent compared with the individual treatment groups (all *p* < 0.001, Figure 3C). Therefore, metformin combined with FuOx induced CRC cell arrest in the HT29, SW480, and SW620 cell lines.

### 3.3. The Combination of Metformin and FuOx Induced Cell Apoptosis

**Figure 4:**
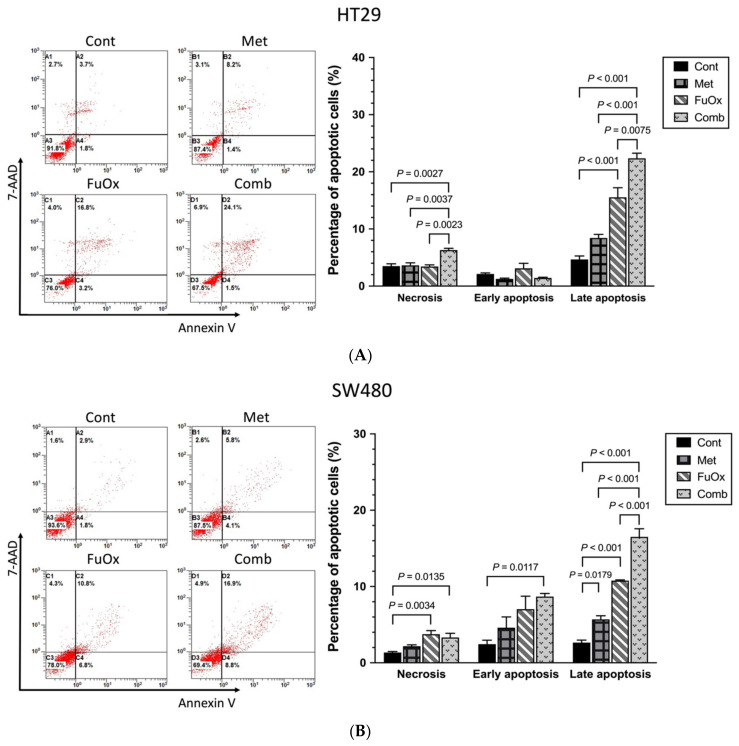

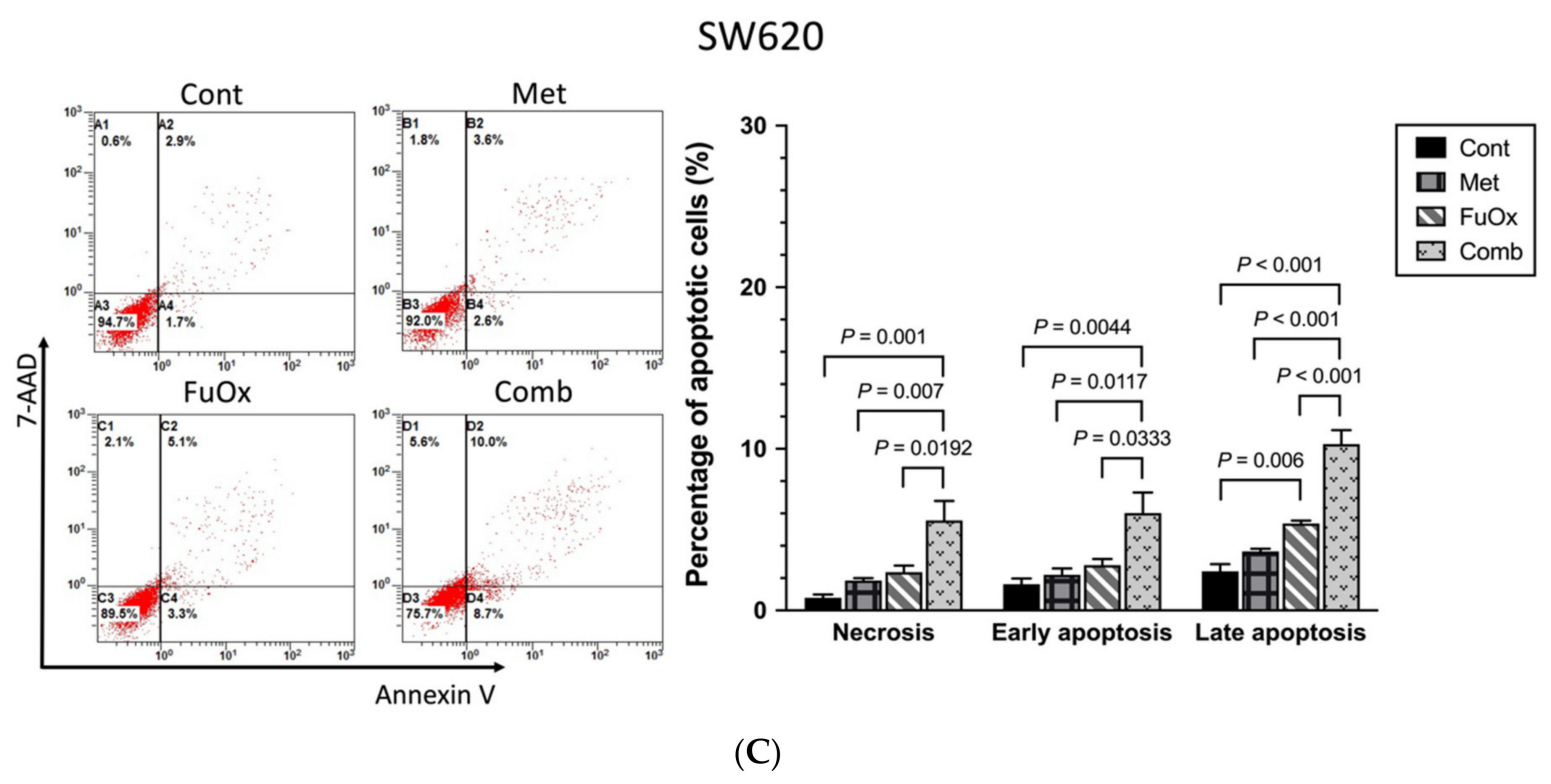
The combination therapy group induced greater apoptosis than metformin alone and FuOx alone groups in HT29, SW480, and SW620 cell lines. CRC cells were analyzed using flow cytometry after 72 h of treatment with metformin (5 mM), FuOx (2 µM), and the combination of metformin and FuOx. Representative flow cytometry dot plots (**left** panel) and histograms (**right** panel) reveal cell apoptosis and quantitative analysis, respectively, for (**A**) HT29, (**B**) SW480, and (**C**) SW620 cell lines. Data are presented as mean ± SEM. Cont, control; Met, metformin; FuOx, 5FU and oxaliplatin; Comb, combination of metformin and FuOx.

To evaluate whether metformin and FuOx influence apoptosis, we analyzed the number of apoptotic cells after different treatments by using the Annexin V/7-AAD staining assay through flow cytometry. The results revealed that metformin combined with FuOx increased the percentage of SW480 and SW620 early apoptotic cells compared with the control treatment (*p* < 0.05, Figure 4B,C). However, this effect was not observed in the HT29 cells (*p* = 0.7196, Figure 4A). Moreover, the percentage of late apoptotic cells increased in the HT29, SW480, and SW620 cells after treatment with metformin or FuOx alone. Metformin significantly increased the effects of FuOx in late apoptosis in the HT29, SW480, and SW620 cells (all *p* < 0.01). These results demonstrate that combined metformin and FuOx promotes more apoptosis of CRC cells.

### 3.4. Metformin Enhanced the Chemosensitivity of CRC Cells to FuOx through Mitochondria-Related Proteins Mcl-1 and Bim

**Figure 5:**
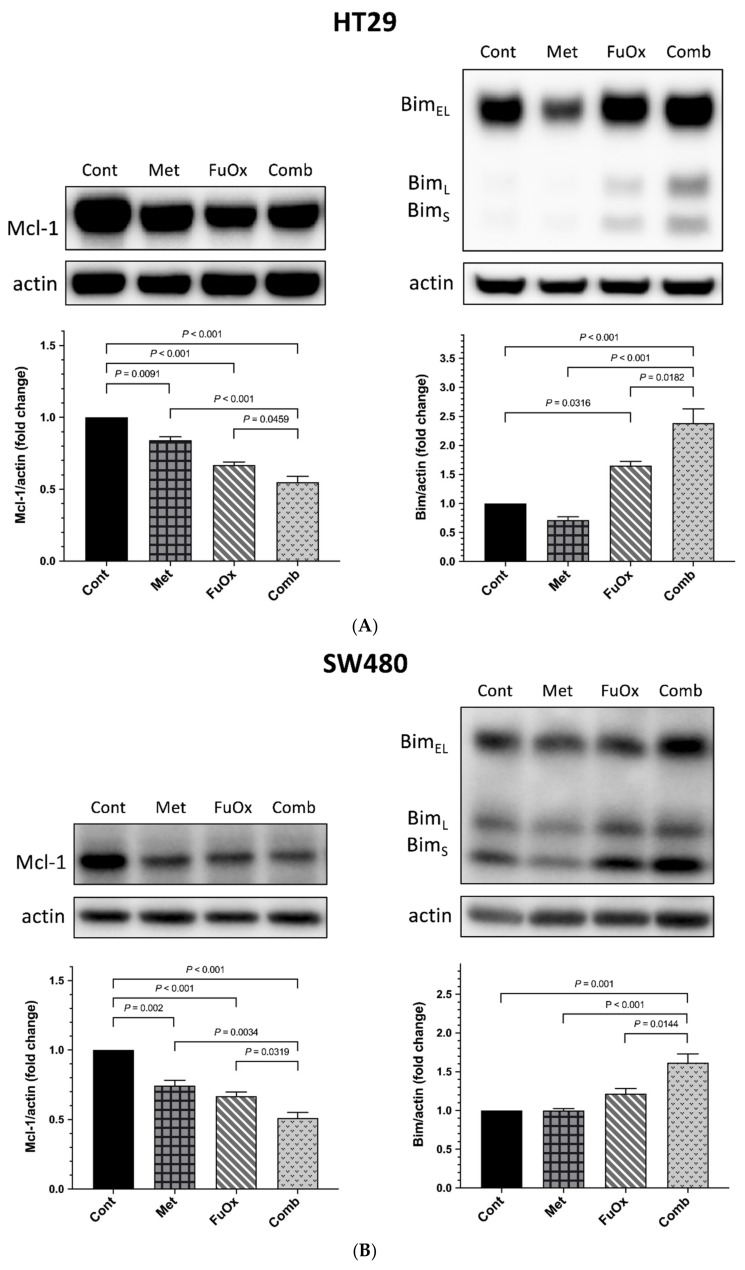

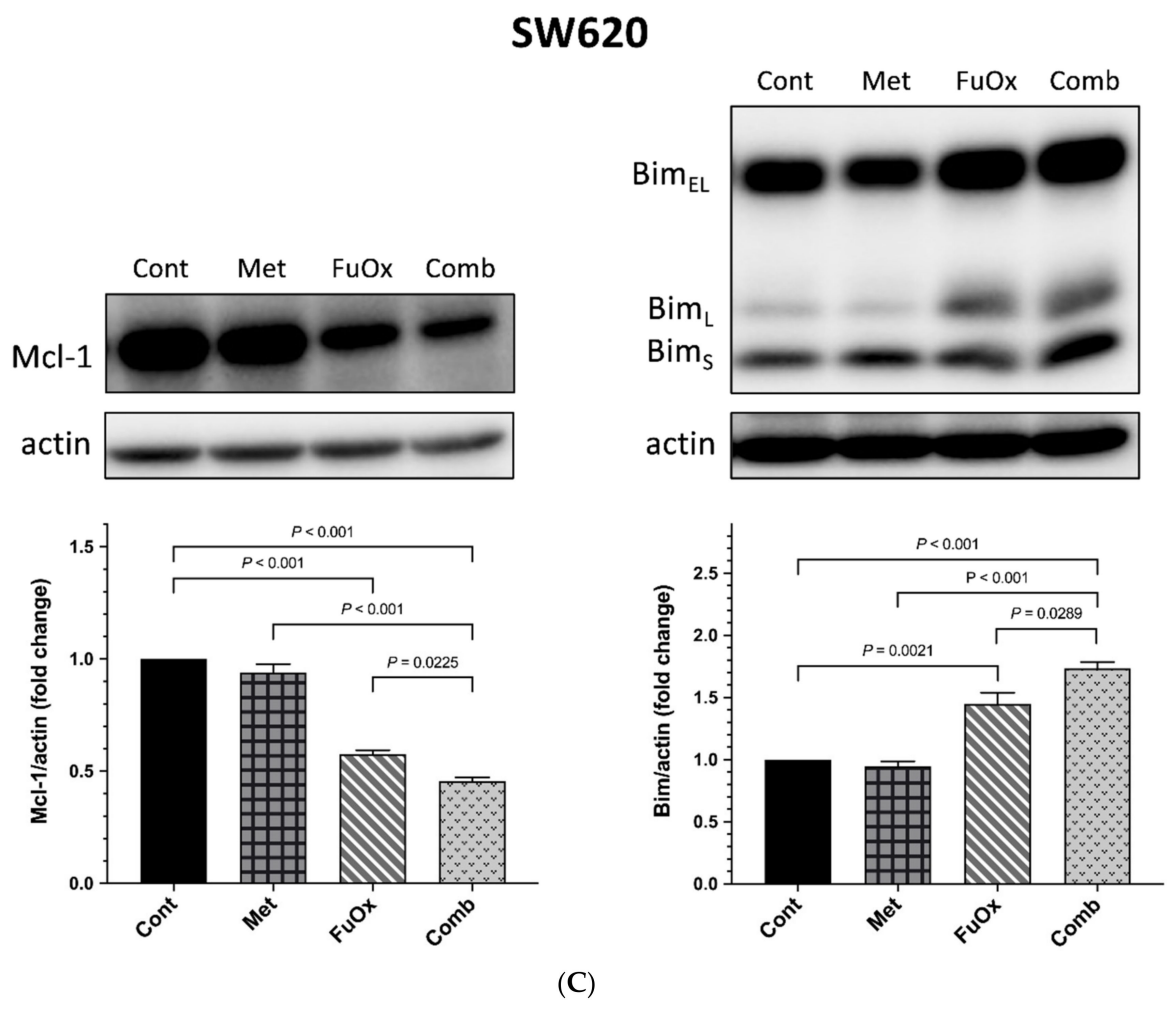
Metformin enhanced the expression of FuOx by modulating Mcl-1 and Bim in HT29, SW480, and SW620 cell lines. Mcl-1 and Bim protein levels in (**A**) HT29, (**B**) SW480, and (**C**) SW620 cell lines were analyzed through Western blot after 72 h treatment with metformin (5 mM), FuOx (2 µM), and the combination of metformin and FuOx. Data are presented as mean ± SEM. Cont, control; Met, metformin; FuOx, 5FU and oxaliplatin; Comb, combination of metformin and FuOx.

To further extend our findings, we determined the levels of the proapoptotic protein Bim and the antiapoptotic protein Mcl-1 through Western blotting. Bim has multiple isoforms; all of these isoforms are involved in proapoptosis. The expression of Mcl-1 in the HT29, SW480, and SW620 cells in the combination group was significantly reduced relative to that in the control, metformin alone, and FuOx alone groups (all *p* < 0.05, Figure 5). In addition, the Bim level was notably higher in the HT29, SW480, and SW620 cells after treatment with the combination of metformin and FuOx (all *p* < 0.05). The results demonstrated that treatment with the combination of metformin and FuOx downregulated the expression of Mcl-1 and notably upregulated the expression of Bim in the CRC cells.

### 3.5. The Tumor Growth in BALB/c Nude Mice Was Suppressed after Treatment with Metformin Combined with FuOx

**Figure 6:**
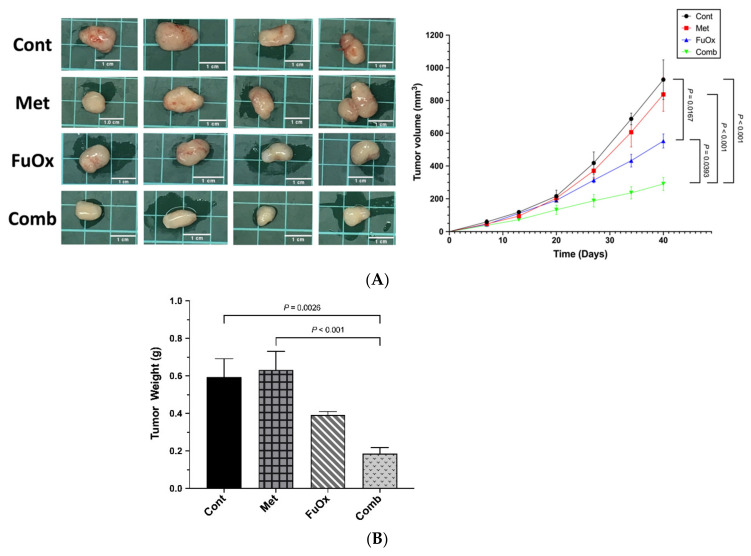
The combination of FuOx and metformin decreased tumor volume and tumor weight in BALB/c nude mice. HT29 was injected subcutaneously into the right flank of nude mice. Treatment was started 7 days after HT29 cell injection. Metformin (150 mg/kg) dissolved in drinking water was orally administered daily. The mice were injected with 6 mg/kg intraperitoneal oxaliplatin and 2 h later with 50 mg/kg 5-FU and 90 mg/kg leucovorin once a week for 4 weeks. (**A**) The tumor volume was measured three times per week; (**B**) the tumor weight was recorded after sacrificing at day 40. Data are presented as mean ± SEM. Cont, control; Met, metformin; FuOx, 5FU and oxaliplatin; Comb, combination of metformin and FuOx.

To validate in vivo that metformin could increase the antitumor effects of FuOx, we subcutaneously injected HT29 cells into the right flanks of nude mice. After 7 days, therapy was commenced. As presented in Figure 6A, the tumor growth rates in the FuOx alone and combination groups were notably reduced compared with the control group (*p* < 0.05). Furthermore, compared with the metformin and FuOx alone groups, the metformin and FuOx combination group demonstrated more prominent inhibition of the growth of tumors (*p* < 0.05). The tumor weight reduction in the combination group was notable relative to those of the untreated control and metformin alone groups (*p* < 0.01, Figure 6B). However, tumor weight did not statistically differ between the FuOx alone and combination groups (*p* = 0.0682). We observed that the combined treatment of metformin and FuOx obviously suppressed HT29 tumor cell growth in BALB/c nude mice.

## 4. Discussion

According to the Taiwan Cancer Registry annual report of 2019, among all malignancies in Taiwan, CRC is ranked first in terms of the incidence rate (17,302 cases; 14.3% of total new cases) and third in terms of the mortality rate (6436 cases; 12.8% of total death cases). From 2009 to 2019, the incidence of CRC cases increased from 12,488 to 17,302, and mortality increased from 4531 to 6436. Furthermore, the age at which patients develop CRC has gradually decreased, with the number of CRC cases involving patients aged <50 years increasing from 1503 to 2136. A growing body of research has reported that hyperglycemia is a key risk factor for CRC development, with hyperglycemia being closely correlated with the progression, incidence, and prognosis of CRC [27,28]. Our previous study revealed that high blood sugar levels significantly enhanced chemoresistance in patients with stage III CRC undergoing adjuvant FOLFOX6 chemotherapy [29]. Therefore, researchers are actively searching for more effective treatment strategies to combat CRC for improving survival rates.

Metformin is a Food and Drug Administration–approved first-line drug for patients with T2DM [30]. In addition to increasing patients’ insulin sensitivity, metformin reduces hepatic gluconeogenesis and increases glucose consumption of the muscles, thereby decreasing blood glucose levels [31]. Metformin has been used as monotherapy and in combination with other antidiabetic drugs. Metformin does not cause hypoglycemia because it does not increase insulin secretion, which renders it a more favorable option than other oral antidiabetic medications. Metformin has been reported to be effective in treating not only hyperglycemia but also other diseases, including polycystic ovarian syndrome, cardiovascular disease, and cancer [32]. In antitumor treatment, metformin mainly inhibits mammalian target of rapamycin and its downstream target through modulating mitochondrial functions and the expression of the PI3k/Akt pathway, thereby curbing tumor cell survival [33].

Evidence has indicated that metformin has a synergistic effect when used in combination with other chemotherapeutic agents [34,35,36]. Hirsch et al., demonstrated that the combination of doxorubicin and metformin significantly reduced tumor volume and the recurrence of breast cancer in a mouse model [37]. Khader et al., revealed that irinotecan combined with metformin enhanced the cytotoxicity of HCT116 and SW480 cells and induced cell cycle arrest in the G1 and S phases [38]. Nangia-Makker et al., reported that metformin in combination with 5-FU and oxaliplatin could synergistically inhibit the proliferation of chemoresistant colon cancer cells using in vitro and in vivo xenograft models [39]. These findings demonstrate that metformin is a potential effective adjunctive agent in cancer treatment.

Studies have reported that metformin has a significant apoptosis-inducing effect on *p53* mutant cell lines [40,41]. The CRC cell lines HT29, SW480, and SW620 with *p53* mutations were used in the present study. As discussed, metformin has the potential to increase the sensitivity of cancer cells to chemotherapy drugs. Evidence has revealed that metformin can enhance the treatment effects of 5-FU on various cancers, and it can significantly inhibit the growth of cancer cells in vitro and in vivo [24,42,43,44]. Studies have demonstrated that the combination of metformin and 5-FU induces a more favorable response than 5-FU alone does [45,46]. Therefore, in our study, we selected two chemotherapeutic agents of 5-FU and oxaliplatin in conjunction with metformin to mimic chemotherapy regimens used in clinical settings.

Zhang et al., demonstrated that the combination of cisplatin and metformin affected mitochondrial function in CRC cells by lowering mitochondrial membrane potential (MMP) and inducing reactive oxygen species (ROS) production, thereby promoting apoptosis through the PI3K/Akt pathway [47]. Chen et al., also reported that metformin induces ROS-dependent apoptosis in HCT116 CRC cells through the MCl-1/Bim/Bak signaling pathway [48]. Several studies have further revealed that Mcl-1 amplification is a crucial factor in chemoresistance, which is associated with poor prognosis in patients with cancer [49,50]. The overexpression of Mcl-1 maintained cell survival and contributed to resistance to chemotherapeutic response in CRC. In the present study, we observed that metformin enhanced the chemosensitivity to FuOx by inhibiting Mcl-1 protein expression and increasing Bim protein expression.

In clinical practice, the recommended daily dosage of metformin for adults is 500–2000 mg. The dosage we used for our BALB/c nude mice was calculated using a previously published dosage conversion formula [51]. According to the formula, 150 mg/kg/day for mice is equivalent to an adult human dosage of 12.162 mg/kg/day, which is approximately 600–1000 mg for 50–80 kg adults. We used this dosage for the mice in this experiment to mimic clinical practice as closely as possible.

This study has a few limitations. First, the Mcl-1/Bim signaling pathway was not completely elucidated in our study, although Mcl-1 and Bim are involved in initiating tumor apoptosis. Therefore, the mechanism underlying the combined effect of metformin and FuOx requires further exploration. Second, other studies have reported that metformin can cause mitochondrial dysfunction to preclude the progression of cancer [47,52]. Hence, several mitochondrial function assays, such as those of mitochondrial superoxide production and MMP, should be conducted to better understand the association between FuOx and metformin and mitochondria. Third, invasion assay should be performed to comprehensively assess the therapeutic effect of FuOx combined with metformin.

The aim of the present study was to elucidate the combined effects of metformin and FuOx in CRC cell lines and an animal model. Through a series of in vitro experiments, we obtained results that demonstrated that metformin could enhance the efficacy of FuOx in inhibiting cell proliferation, cell survival, and migration; inducing cell cycle arrest; and promoting apoptosis. Western blot analysis further verified the molecular apoptotic mechanism, which resulted from the underexpression of Mcl-1 proteins and overexpression of Bim proteins.

Through in vivo studies, we further verified the effectiveness of the combination therapy. The data revealed that, after 40 days of treatment, the combination of metformin and FuOx had a more pronounced effect on the inhibition of tumor growth and tumor weight than FuOx or metformin alone. We suggest that when combined with FuOx, metformin potentially acts as an adjunctive agent to eliminate CRC in vitro and in vivo.

## Data Availability

Not applicable.
